# Two coexisting heterozygous frameshift mutations in *PROP1* are responsible for a different phenotype of combined pituitary hormone deficiency

**DOI:** 10.1007/s13353-015-0328-z

**Published:** 2015-11-25

**Authors:** K. Ziemnicka, B. Budny, K. Drobnik, D. Baszko-Błaszyk, M. Stajgis, K. Katulska, R. Waśko, E. Wrotkowska, R. Słomski, M. Ruchała

**Affiliations:** 1 Molecular Endocrinology Laboratory, Department of Endocrinology, Metabolism and Internal Diseases, Poznan University of Medical Sciences, 49 Przybyszewskiego Str., 60-355 Poznan, Poland; 2 Department of General Radiology II, Poznan University of Medical Sciences, Poznan, Poland; 3 Department of Biochemistry and Biotechnology, University of Agriculture, Poznan, Poland; 4 Institute of Human Genetics, Polish Academy of Sciences, Poznan, Poland

**Keywords:** CPHD, Combined pituitary hormone deficiency, *PROP1*

## Abstract

The role of genetic background in childhood-onset combined pituitary hormone deficiency (CPHD) has been extensively studied. The major contributors are the *PROP1*, *POU1F1*, *LHX3*, *LHX4* and *HESX1* genes coding transcription factors implicated in pituitary organogenesis. The clinical consequences of mutations encompass impaired synthesis of a growth hormone (GH) and one or more concurrent pituitary hormones (i.e. LH, FSH, TSH, PRL). Manifestation of the disorder may vary due to various mutation impacts on the final gene products or an influence of environmental factors during pituitary organogenesis. We describe the clinical and molecular characteristics of two brothers aged 47 and 39 years presenting an uncommon manifestation of congenital hypopituitarism. Sequencing of the *PROP1*, *POU1F1*, *LHX3*, *LHX4* and *HESX1* genes was performed to confirm the genetic origin of the disorder. A compound heterozygosity in the *PROP1* gene has been identified for both probands. The first change represents a mutational hot spot (c.150delA, p.R53fsX164), whereas the second is a novel alteration (p.R112X) that leads to protein disruption. Based on precise genetic diagnosis, an in silico prediction of a p.R112X mutation on protein architecture was performed. The resulting clinical phenotype was surprisingly distinct compared to most patients with genetic alterations in *PROP1* reported in the current literature. This may be caused by a residual activity of a newly identified p.R112X protein that preserves over 70 % of the homeodomain structure. This examination may confirm a key role of a DNA-binding homeodomain in maintaining *PROP1* functionality and suggests a conceivable explanation of an unusual phenotype.

## Introduction

Intensive studies of pituitary organogenesis revealed the indisputable role of transcription factors genes defects in the development of familial combined pituitary hormone deficiency (CPHD). Several mutations have been described within *HESX1*, *LHX3*, *LHX4*, *PROP1*, *POU1F1*, *PTX* and other genes (Romero et al. 2011). Small recurring deletions in the *PROP1* gene coding transcription factor taking part in the regulation of pituitary organogenesis are the most common cause of genetically determined CPHD (Böttner et al. 2004; Lebl et al. 2005; Lemos et al. 2006; Voutetakis et al. 2004a). The gene is composed of three highly conserved exons that code for the protein of 226 amino acids residues, retaining the abilities of DNA binding and transcriptional activation (Sornson et al. 1996). Most of these mutations are located within the second exon of the *PROP1* gene, although there are some variations of mutations patterns reported for different populations (Kim et al. 2003; McLennan et al. 2003). In more than half of familial CPHD cases, *PROP1* gene mutations have been identified (Deladoëy et al. 1999). The most common mutation is 301-302delAG and the second is 150delA; other mutations are less frequent. Noticeably lower common frequency of these mutational hot spots was detected among sporadic patients (Turton et al. 2005), supporting the theory of a founder effect mechanism instead of its de novo nature.

*PROP1* gene mutations lead to hypopituitarism that is characterised by insufficiency of growth hormone (GH), luteinising hormone (LH), follicle-stimulating hormone (FSH) and thyroid-stimulating hormone (TSH) synthesis and release. Usually, the first noted symptom is growth failure, and the observed onset encompasses 2 to 8 years of age. The manifestation of central hypothyroidism appears in varying degrees. Typically, in the second decade of life, no sexual maturation is evident and the genitals remain infantile. In some cases of CPHD, usually in the second or even third decades of life, secondary adrenocortical failure develops or a selective DHEA deficiency is seen with normal ACTH–cortisol axis function. In parallel, prolactin deficiency might be present (Flück et al. 1998; Mody et al. 2002; Voutetakis et al. 2001).

The aim of the present work was to evaluate the genetic background of CPHD in two brothers with unusual manifestation of a disorder and to describe the uncommon phenotype.

## Patients and methods

### Patients

Patient 1 was born to parents of normal height after a full-term pregnancy without any complications; his physical and mental development was normal in the first 5 years of life without marked growth disturbances [−1.0 standard deviation (SD)]. When the patient was 6 years old, his parents noted growth deficiency, pallor of the skin and marked facial oedema. The paediatrician suspected hypothyroidism that was seemingly supported by hypercholesterolaemia and lowered serum thyroid hormone concentrations: total T4 was 0.8 μg/dL (normal range: 4–12) and total T3 was 76 ng/dL (normal range: 80–200). At that time, analysis of the TSH concentration was not available. The paediatrician commenced levothyroxine therapy. Following this treatment, the patient showed improvement and oedema regressed; however, the linear growth rate was deficient (−2.7 SD). His mental development was normal and he had no learning difficulties at school. At the late age of puberty, signs of sexual maturation were not present and the patient was referred to the Department of Endocrinology, where, based on the hormonal test results, a diagnosis of childhood-onset CPHD (deficiency of GH, TSH and LH/FSH) was made. At that time, computed tomography (CT) scan of the pituitary area revealed the presence of a hyperdense mass (the density of which did not increase after contrast administration) in a markedly enlarged sella. That lesion was surrounded by fluid. Magnetic resonance imaging (MRI) performed during the next medical evaluation (12 years later) showed remnants of the pituitary gland with a height of about 5–6 mm and a hypodense lesion (about 6 mm in diameter) between the anterior and posterior lobes not enhanced with contrast and localised at the left side of the gland. The patient was treated with long-acting testosterone derivatives and levothyroxine (treatment with growth hormone was not available at that moment). He felt well and his growth rate slowly advanced. even in the third decade of life. At the age of 34 years, radiograms of the hands, hips and femurs revealed that epiphyseal fusion did not occur.

Patient 2 was born 8 years after patient 1. At birth, his weight, length and body proportions were normal. His physical and mental development, in the first four years of life, had a normal course. When he was 4 years old, a mild slowing of the growth rate was noticed (−1.5 SD); the stunted growth was more evident when the patient started his school education at the age of 7 years (−2.0 SD). He was referred to the paediatrician who, in the case history, described skin dryness, puffiness of the face and extremities, increased cholesterol level in the blood and diagnosed hypothyroidism, suspecting a similar problem as in the case of his older brother. The detailed information about his hormone levels at that moment was not available to us. The patient was then treated with levothyroxine and anabolic steroid medication (methandienone, 5 mg daily) to stimulate the stunted growth. This therapy was irregularly continued for several years and resulted in improvement of the patient; however, signs of sexual maturation did not appear and his growth was still deficient (−2.1 SD). At 15 years of age, he was intermittently treated with testosterone enanthate 100 mg intra-muscularly every 2 weeks and 100 μg of levothyroxine daily for several years, resulting in slow linear growth rate (even past the age of 20 years), but only slight sexual improvement.

Both brothers were treated and remained under the control of a local endocrinologist. The serum hormone levels were re-evaluated at ages of 47 years for patient 1 and 39 years for patient 2, when they were referred to the Department of Endocrinology, Metabolism and Internal Diseases, Poznan University of Medical Sciences, Poland to establish the treatment direction after a long period of irregular hormone replacement therapy.

### Hormonal assays and tests

On the patients’ re-evaluations, the basal and stimulated serum GH levels were measured by an immunoradiometric assay (IRMA; DSL Inc., Webster, TX, USA). The GH response in each patient was assessed during insulin-induced hypoglycaemia (0,1 IU/kg; Actrapid insulin, Novo Nordisk, Denmark). The serum IGF-1 concentration was estimated using SM-C-RIA-CT kits (BioSource, Nivelles, Belgium). TSH, fT4, prolactin, cortisol, DHEA-S, LH, FSH and insulin were measured by an immunochemiluminometric assay (ICMA; Roche Diagnostics Elecsys 2010, Basel, Switzerland). The serum ACTH level was assessed by immunoradiometric assay (B.R.A.H.M.S., Hennigsdorf, Germany) and free testosterone was evaluated using RIA kits (DPC, Monroe, LA, USA). The pituitary–adrenal axis was evaluated using insulin-induced hypoglycaemia according to standard procedures, considering normal adrenal response when the cortisol level was over 550 nmol/L. Beside basic levels, TSH and prolactin were also estimated in the TRH stimulation test (200 μg Protirelin i.v., Merck, Darmstadt, Germany). The LH/FSH response was investigated using the GnRH test (100 μg GnRH, Ferring, Germany).

### Radiological imaging

Pituitary MRI was performed using the spin-echo technique in T1-weighted coronal and sagittal scans. Patient 1’s sagittal T1-weighted pituitary images were performed with a Siemens MAGNETOM Avanto and acquired using multi-slice spin-echo pulse sequences with parameters of 800/15/4 (TR/TE/excitations), 3-mm slice thickness with 1-mm interslice gap, 256 × 256 acquisition matrix and a 24-cm field of view. Scans for patient 2 were performed with a Siemens 1T MAGNETOM Impact using multi-slice spin-echo pulse sequences with 500/12/4 (TR/TE/excitations), 3-mm slice thickness with 1-mm interslice gap and a 184 × 210 acquisition matrix. Coronal T1-weighted images were also obtained with a field of view of 20 cm for both patients. Radiograms of the skull were evaluated in the sagittal and coronal planes, with the sella turcica length measurement expressed as a distance from the tuberculum sellae to the posterior clinoid (Andredaki et al. 2007).

### Molecular screening of CPHD genes

Blood samples were collected from both brothers. Genomic DNA was extracted from peripheral blood leukocytes according to standard procedures (GTC method). Mutation scanning was carried out by polymerase chain reaction (PCR) and following sequencing of the PCR products. The entire coding sequence of *PROP1*, *POU1F1*, *LHX3* and *LHX4* was amplified, as well as neighbouring exons’ intronic sequences (at least 50 nucleotides of each intron–exon boundary). The sequence of *PROP1* primers for PCR reaction was used according to those previously published: for the first exon by Deladoëy et al. (1999) and for the second exon by Rosenbloom et al. (1999). For the third exon, a nested PCR was utilised with a new primer pair that was designed on the basis of the *PROP1* genomic sequence. Primer sequences for *POU1F1*, *LHX3*, *LHX4* and *HESX1* were designed using the Primer3 algorithm (http://bioinfo.ut.ee/primer3-0.4.0/). Genomic sequences for all mentioned genes were recovered from GeeBank. All primer sequences for PCR are given in Table 1.

**Table 1:** Primer sequences used for *PROP1*, *POU1F1*, *LHX3*, *LHX4* and *HESX1* screening

Genomic sequence: hg19_chr5:177418235-177424243_rev			
*PROP1*	Left primer	Right primer	Product size
Exon1	TTCAGAGACAGAGTCCCAGA	CTCCTAACCTTCTTCATGGA	326
Exon2	TGGTCCAGCACCGAGAG	TGCCCAACATTCTATGATAGC	366
Exon3a	TCTGGCCATGCTGGAGAAG	TTCTAATCGCTGAGCTGAC	579
Exon3b (nested)	CCCTGCACCTCTTGTCATTGGAGT	AAGCCACCCCATTTTCTTGTCTTT	492
Genomic sequence: hg19_chr3:87307782-87326737_rev			
*POU1F1*	Left primer	Right primer	Product size
Exon1	ATTGAATCGGCCCTTTGAG	GGGTAAAATGAAAGATGCAAAG	334
Exon2	CCTACTCGTCAGAGAACTTACCC	TCTGATCACAATTCTTTCAGGC	358
Exon3	TGGGCTAAGTCAGGCAAAAC	TCCATAACGACTAACTACGTCCAC	454
Exon4	CAGATTTGTGTGACAATGAACC	AAAACCCCTCAAACCTCCTG	342
Exon5	CATTCCAACAAAAGTAAAGTGAGG	CTGGGATTATAGGCACCCAC	538
Exon6	TGTCCTGCAAGTGTGTTCAG	TGTGAGAAAGAGAGCGGGAG	632
Genomic sequence: hg19_chr9:139087095-139097955_rev			
*LHX3*	Left primer	Right primer	Product size
Exon1	CTCCAGGGGACGCTGAC	ACTTTCTTTGCCTGGCCG	234
Exon2	AAGGTGGCTTCACTGCCTC	CTTGGTGATTGTGAGGGGAG	310
Exon3	GCTCGGGGCGAAATGAG	GGAGAGAATTTCCCCGGAC	354
Exon4/5	CCTTCCGAGAAGCCTGTG	TCCATGGGAAATTCAGATCC	642
Exon6	CAGGATGGGACTCTGAGGG	CCTGGCCCCACTTCCTC	580
Genomic sequence: hg19_chr1:180198432-18024518			
*LHX4*	Left primer	Right primer	Product size
Exon1	AGCTAGAGCGAGAGAGCGAG	CCCTGTGACCGCCTCTG	268
Exon2	TGGTTAGCAGGGCTGTGTG	CTCACTGCTTGGGGAGAGG	289
Exon3	GAAGCCAGATCCCTTGCTC	GAGAAGGGCACCTCAGGC	335
Exon4	AGGGTGTGGGAGGAGGC	TCACTCAGGATACCTTCCACC	295
Exon5	GCTTTGGGTTTGTGGTGG	TCCTGAGTGCCAGGGATTAC	302
Exon6	GGGACCATCAGAGTCCTGG	TTCGATCCTTAAAAGGCAAG	535
Genomic sequence: hg19_chr3:57230943-57235280_rev			
*HESX*	Left primer	Right primer	Product size
Exon1	CCTATACACGTGGGGCAGAG	TGAAATAAAGGGCAAATTAAACAC	290
Exon2	TCCTGAAACTACCTCTATAGAACTTTG	TGCTCAACTTGGTGTCAATTAAAG	339
Exon3/4	AGACTACCATATTTTAACAATTTCCAG	CACTGATTCTTCATGCTCTGC	498

 Amplifications were conducted with the use of the FailSafe™ PCR PreMix J System (Epicentre, Illumina) and processed through 35 cycles (30 s at 94 °C, 30 s at 62 °C and 45 s at 72 °C). All fragments were then subjected to Sanger sequencing on both strands. Capillary sequencing was conducted with the use of BigDye chemistry version 3.1 on an ABI 3130xl DNA Analyzer, according to the manufacturer’s instructions (Applied Biosystems, Foster City, CA, USA). Samples were processed through 30 cycles of amplification consisting of 30 s at 94 °C, 30 s at 60 °C and 45 s at 72 °C. The final step was lengthened to 7 min.

 Analysis of sequence tracks was achieved with the use of CodonCode Aligner software version 4.0.4 (http://www.codoncode.com). Patients were also screened for structural rearrangements in the *PROP1*, *POU1F1*, *LHX3*, *LHX4*, *HESX1* and, additionally, *GH1* and *GHRHR* genes using the multiplex ligation-dependent probe amplification (MLPA) method. MLPA reaction was performed using SALSA MLPA P216 Growth Hormone Deficiency mix −1 from MRC-Holland (Netherlands), according to the manufacturer’s instructions. This kit contains probes for all exons of the mentioned genes except for exon 2 of *GH1*, exon 5 of *POU1F1* and exon 8 of *GHRHR*. The amount of genomic DNA used for the MLPA analysis was 200 ng. PCR products were separated on an ABI 3130xl Capillary Sequencer (Applied Biosystems). Data were normalised intra-sample by dividing the peak area of each probe’s amplification product by the total area of only the reference probes in the probemix. Intra-sample normalisation was achieved by dividing the intra-normalised probe ratio of all reference samples. Data normalisation was performed within one experiment (MRC-Holland). Peak areas in the range 0.7–1.3 are normal, below 0.7 is considered as one copy deletion and above 1.3 for duplication. Each MLPA analysis included several control samples (DNA from healthy individuals).

### Bioinformatics approaches

The identified mutations were checked for correctness of position assessment with the use of the MutationTaster2 program (Schwarz et al. 2010). By employing Bayes classifiers, MutationTaster2 facilitates prediction of the pathogenicity for an alteration, possible impact of nonsense-mediated decay (NMD) and search for reported variations in the region of novel mutation within selected databases [dbSNP/TGP/HGMD(public)/ClinVar]. Computation of the *PROP1* homeodomain was conveyed with the use of SWISS-MODEL (Schwede et al. 2003) and Phyre2 (Kelley and Sternberg 2009), including ten flanking amino acids from both ends. The results were than subsequently compared and evaluated. Confidence in the models was as follows: 78 residues (98 %) modelled at >90 % accuracy for the reference homeodomain and 50 residues (96 %) modelled at >90 % accuracy for the p.R112X homeodomain. The protein structures (pdb files) were subjected to molecular graphics and further analyses with the use of the UCSF Chimera package (Pettersen et al. 2004; Yang et al. 2012).

## Results

Both patients were admitted to the Department of Endocrinology, Poznan University of Medical Sciences after irregular therapy with L-thyroxine and testosterone enanthate for hormonal re-evaluation and imaging study. On physical examination of the 47-year-old patient 1, his height was 152 cm (−4.0 SD), body proportions normal and his arm span measured 152 cm. He was obese [body mass index (BMI) 30.2 kg/m^2^]. His head circumference (measured just above the ears and eyebrows level) was 58 cm (75th percentile), face was pale and bloated with puffed upper eyelids and cheeks, and his nose was broad. His skin was dry, muscles poorly developed and subcutaneous fat tissue was abundant, especially on the abdomen obscuring the waist; fat pads were also present in the supraclavicular areas. There was no beard growth and no body hair, the axillary and pubic and scrotum hair were scanty. His genitals were underdeveloped, the penis and testicles of child-like proportions and his scrotum was only slightly pigmented (Tanner stage 2). The results of the hormone assays are listed in Table 2.

**Table 2:** Individual blood hormone values in the two studied patients before and after stimulation tests performed during the last hospitalisation (patient 1 at age 47 years and patient 2 at age 39 years)

Hormone	Patient 1		Patient 2		Normal range* (basal evaluation)
	Basal	Peak	Basal	Peak	
GH (ng/mL)	1.1	1.3	0.5	1.3	0–10
IGF-1 (ng/mL)	23	–	56	–	90–412
TSH (mIU/L)	0.2	2.1	0.14	1.9	0.27–4.2
fT4 (pmol/L)	6.5	–	7.1	–	11.5–21.0
LH (mIU/mL)	0.1	1.6	0.1	1.8	1.5–9.0
FSH (mIU/mL)	0.1	1.9	0.1	1.7	1.5–12.5
Testosterone (pg/mL)	2,3	–	4,8	–	5.6–27.0
ACTH (pg/mL)	23.0	59.7	18.1	39.5	0–50
Cortisol (nmol/L)	498.6	715.2	367.1	672.0	220–590
DHEA-S (μg/dL)	95.3	–	55.5	–	100–300
Prolactin (mIU/L)	86.6	109.9	43,3	45.9	85–390

*The cortisol level was evaluated at 8.00 am

Patient 2 was admitted for detailed hormonal investigations when he was 39 years old. On physical examination, his height was 161 cm (−2.88 SD) and his weight was 65 kg. His body proportions were normal. with an arm span of 161 cm. His head circumference (measured as above) was 59 cm (over the 90th percentile), and the patient did not present child-like proportions between the vault and face; also, his nose was broad and well developed. His skin was dry, and a slight puffiness was seen on his face and dorsum of the hands. There was no beard growth and sexual hair was scanty. The size of his genitals was like in the pre-pubertal stage (Tanner stage 1) and bilateral cryptorchidism was present.

Lateral skull radiography performed in both brothers revealed thick calvarial bones. The sella was rounded and of increased dimensions (the sella length in patient 1 was 15 mm and that in patient 2 was 14 mm). The dorsum had a vertical position, with no posterior clinoid processes.

### MRI findings

#### Patient 1

**Fig. 1:**
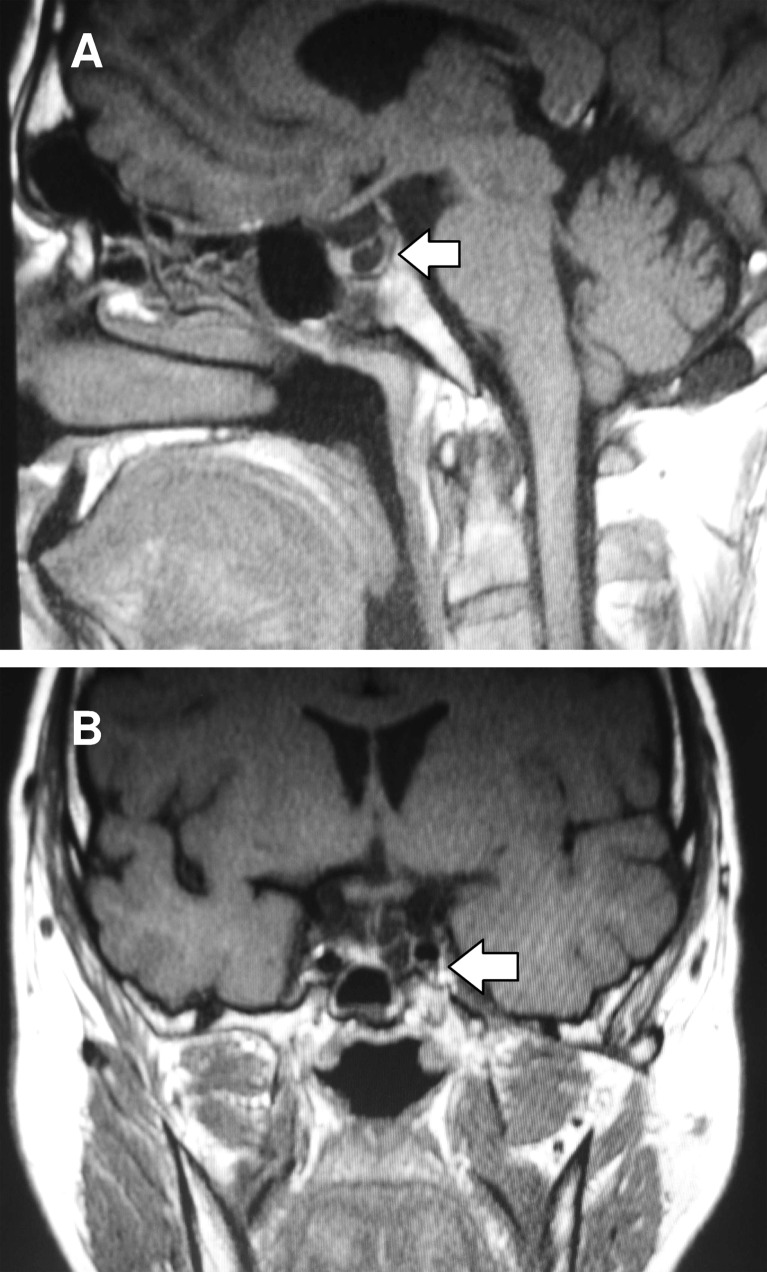
**a** Midline sagittal and **b** coronal magnetic resonance imaging (MRI) sections showing hypoplasia of the pituitary anterior lobe and presence of an anterior lobe cyst in patient 1

Pituitary scans revealed a discrete deepening of the sella and reduced mass of the anterior lobe due to the presence of a cyst on the left side with dimensions 6 × 5 × 7 mm. All pituitary masses had dimensions as follows: height 5–6 mm, width (coronal plane) 13 mm and anteroposterior 12 mm. There was no posterior lobe ectopy or pituitary stalk interruption, although the stalk appeared to be thin (1–2 mm) (Fig. 1).

#### Patient 2

**Fig. 2:**
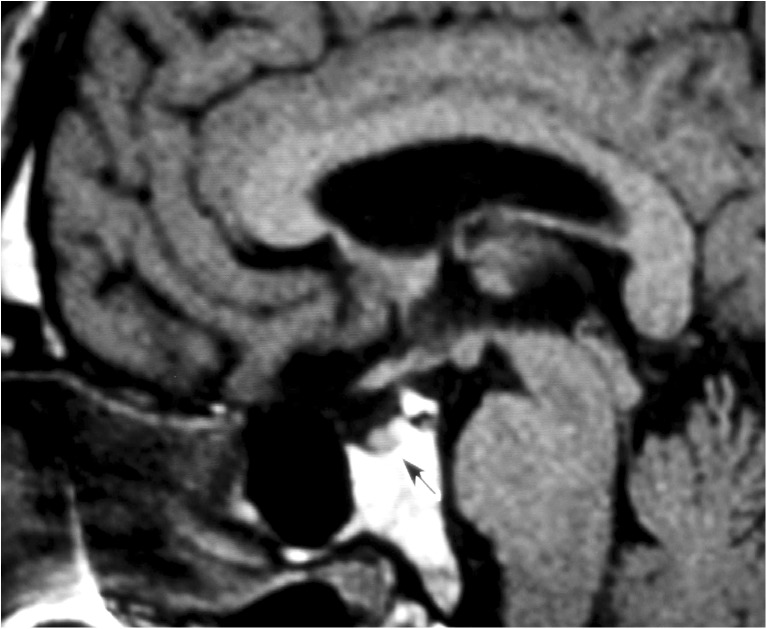
Midline sagittal MRI section showing hypoplasia of the anterior pituitary lobe in patient 2. The *arrow* indicates the small pituitary gland

MRI scans revealed pituitary hypoplasia with height 2 mm, width (coronal plane) 11–12 mm and anteroposterior dimension 10 mm. Like in patient 1, there was no dislocation of the posterior lobe and no interruption of the pituitary stalk (Fig. 2).

### Hormonal studies

Hormonal assays (Table 1) in both patients showed low serum GH level that did not rise during insulin-induced hypoglycaemia and, also, low IGF-1 concentration. The serum-free thyroxine (fT4) level was low, with TSH concentrations below 0.3 mIU/L and poor response during the TRH-TSH test was evident. Serum LH and FSH levels were undetectable and serum-free testosterone was also very low. Serum prolactin was low in patient 2 and normal in patient 1, and, in both cases, only slightly raised in the TRH stimulation test. Plasma ACTH was in the normal range and serum cortisol was normal, whereas serum DHEA was below the normal range.

### Genetic analyses

**Fig. 3:**
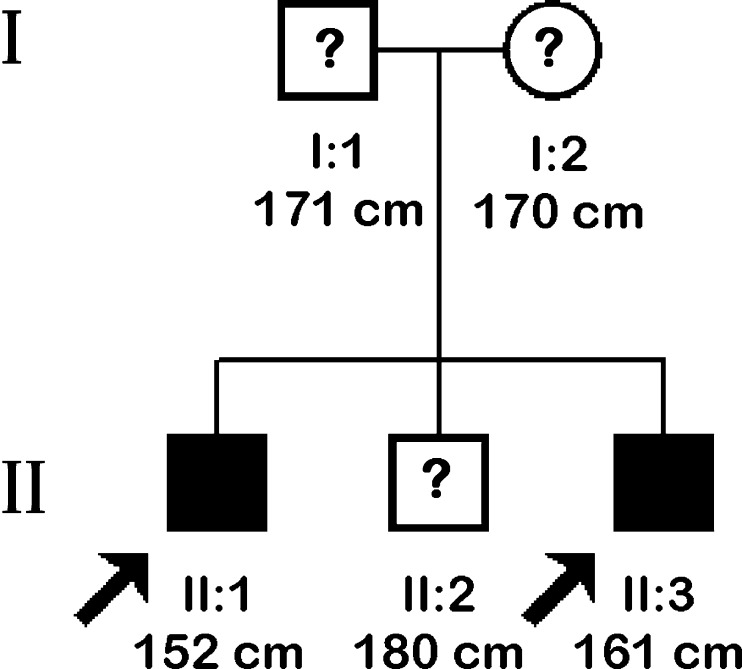
Abbreviated pedigree of the family with *PROP1* gene mutations. The *arrows* indicate the individuals examined in this study. Affected males are denoted by black squares (II:1, II:3), while asymptomatic obligatory heterozygotes (I:1, I:2) and an individual with unknown genetic status (II:2) are depicted by question marks

**Fig. 4:**
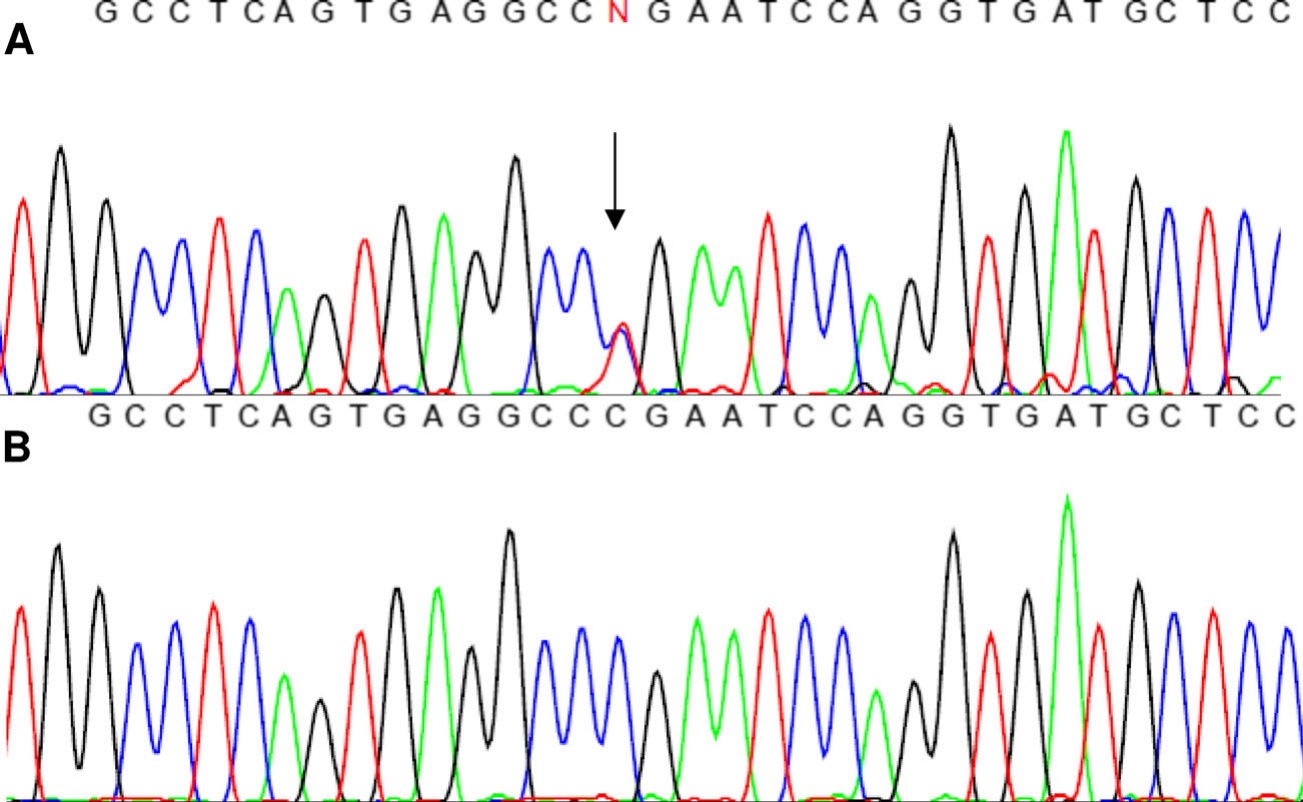
**a** Sequence of the second exon of the *PROP1* gene showing the c.334C>T mutation (position of nucleotide change is indicated by an *arrow*) and **b** sequence of a healthy individual as a normal control

**Fig. 5:**
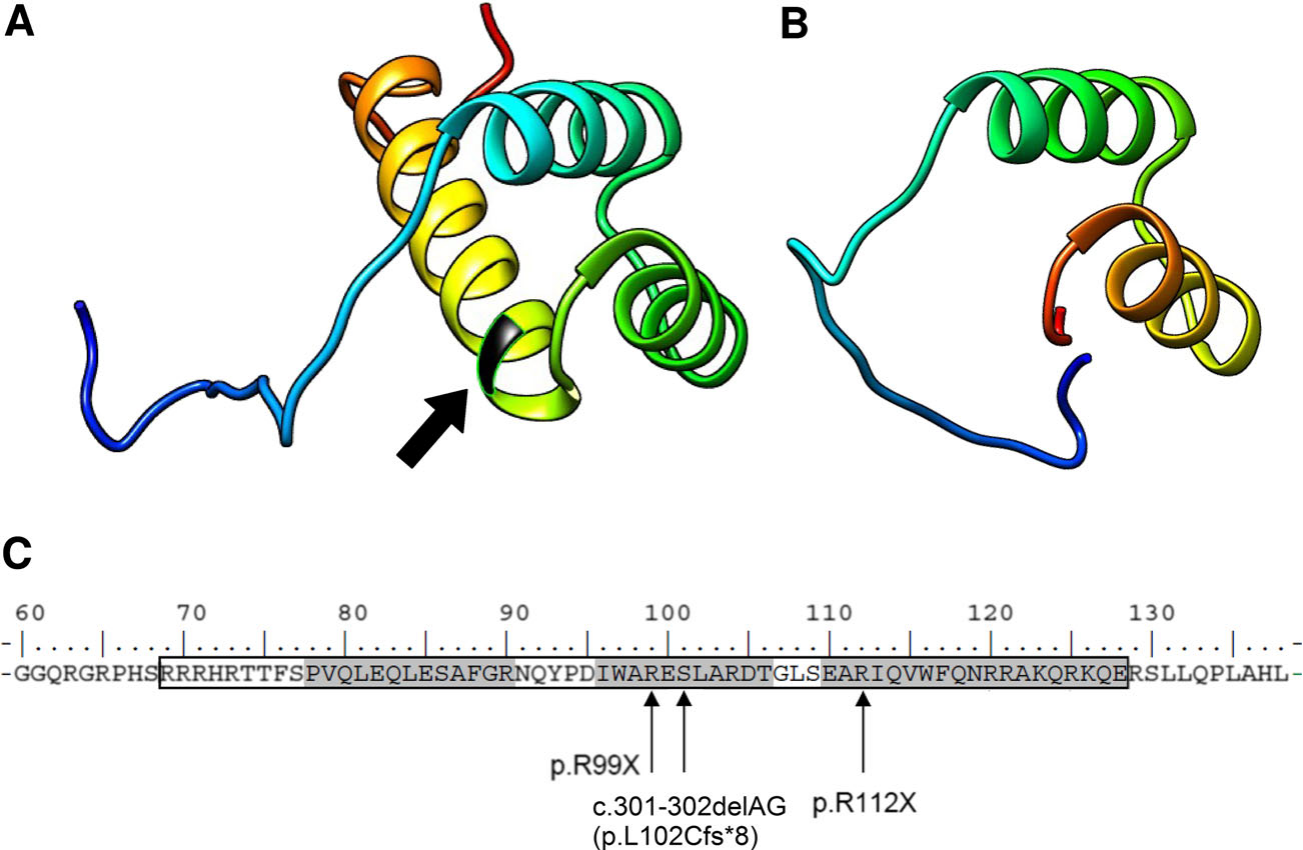
Modelling of the *PROP1* homeodomain (AA 69–128, *framed*). **a** Structural model of the *PROP1* homeodomain with depicted position of 112 arginine (*arrow*). **b** Prediction of the effect of R112X on the structure, revealing the lack of the third, longest alpha helix folding. **c** A string of the selected region in the *PROP1* protein sequence encompassing the homeodomain (*framed*) with three alpha-helix folding (*shaded*) and marked novel mutation site. The mutations lead to a similar phenotype p.R99X, c.301-302delAG (p.L102Cfs*8, also referred to as p.S109X)

Screening for mutations in CPHD genes revealed a compound heterozygosity that occurred in both brothers in the *PROP1* gene. Two different mutations were identified (c.150delA and novel c.334C>T transition), and no other significant change in the reference sequence was present (non-synonymous or rare polymorphisms were also excluded). With regards to an inconspicuous phenotype of the parents and another brother (they did not give consent for genetic study, although according to information provided by the patients, their mother’s height was 170 cm (0.66 SD), the father’s height was 171 cm (−1.3 SD) and the other brother’s height was 180 cm (0.16 SD); the abbreviated family pedigree is shown in Fig. 3). We inferred that both alleles carrying mutations were transmitted independently, confirming the recessive inheritance of CPHD. The c.150delA deletion results in a frameshift of the coding sequence, starting at codon 53 and further leading to a premature termination signal at codon 164 (p.R53fsX164). The c.334C>T substitution (Fig. 4) is reported for the first time and causes an amino acid change of arginine to the STOP codon (p.R112X) and, therefore, protein truncation as well. An in silico prediction using MutationTaster2 confirmed significant protein alteration (115 AA missing, >50 % of the protein sequence) and possible occurrence of the NMD mechanism. Computation of the *PROP1* homeodomain with the use of SWISS-MODEL and Phyre2 revealed the lack of the third, most distally located and longest alpha helix folding within the DNA-binding homeodomain, whereas the structure of the two other alpha helices remained intact (Fig. 5). The mutation screening of *POU1F1*, *LHX3*, *LHX4* and *HESX1* did not reveal any other concurrent abnormalities within coding sequences. The MLPA examination focusing on the identification of copy number abnormalities for selected genes did not result in finding any alterations.

## Discussion

CPHD caused by *PROP1* gene mutation leads to GH, TSH and gonadotropins deficit, but the course of this disorder may vary, even within a family with the same genetic defect (Flück et al. 1998). The diagnosis of GH deficiency usually precedes those of TSH and LH/FSH, and it happens in more than 80 % of these patients (Deladoëy et al. 1999; Mody et al. 2002). Symptoms of central hypothyroidism and mild growth failure in the first years of life, followed by marked growth deficiency and secondary gonadal insufficiency later in life, prevailed in both of the described patients. Thyroid hormone deficiency was clinically manifested as oedema of the face, neck and extremities, and in biochemical evaluation presented as very low T4 and T3 concentrations and high serum cholesterol. Secondary testicular failure at and past the age of puberty was indicated by the lack of any signs of sexual maturation, no beard or sexual hair growth and infantile genitals with cryptorchidism in one patient. The hormone assays test disclosed very low LH, FSH and testosterone concentrations. Despite a prolonged testosterone replacement therapy, signs of masculinisation appeared to be of limited extent, as the genitals still remained infantile and there was no beard growth. This is typical for most patients with CPHD caused by *PROP1* gene defects, but at some points, the clinical manifestation of CPHD in the studied brothers varied. The head circumferences of both patients reached the 75th and 90th percentiles and were typical for 180-cm-tall males, thus differing from other CPHD cases (Bushby et al. 1992; Rosenbloom et al. 1999). Lateral and frontal skull radiographs revealed thick calvarial bones, which may indicate that marked GH deficiency appeared later. The borderline height of patient 2 in the first two decades of life could also support this explanation. Arroyo et al. (2002) reported a unique case of CPHD associated with normal height and absent puberty caused by a *PROP1* defect (R120C) also located in the third helix of the *PROP1* homeodomain.

Hypothyroidism as a first sign is not a frequent finding in CPHD patients and appears in subjects affected by different genetic defects. Deladoëy et al. reported hypothyroidism at 3 years of life in 20 % (7 cases) of the studied CPHD patients with *PROP1* gene mutations (but the particular *PROP1* defects in these cases were not specified) (Deladoëy et al. 1999). Voutetakis at al. also reported on a neonate with a novel *PROP1* gene mutation (Q83X) coexisting with previously described 296delGA which led to protein truncation (the protein product was restricted to only 82 of the 226 amino acids) and, therefore, absence of the DNA-binding and transactivation domain of *PROP1*. A child affected by these mutations presented prolonged jaundice, early manifested central hypothyroidism and pituitary enlargement (Voutetakis et al. 2004b). Flück at al. reported on a girl affected by the R120C *PROP1* gene mutation who presented with a lack of TSH shortly after birth and GH deficiency gradually appearing with age. The other family members carrying the same mutation did not present such course of CPHD, mainly showing severe growth retardation as a first sign (Flück et al. 1998). The functional studies of p.R120C substitution done by Wu et al. revealed that similarly altered mouse Prop1 factor possessed reduced DNA-binding activity and presented impaired trans-activation ability (Wu et al. 1998). Two young siblings under 2 years of age with 296delGA mutation in the *PROP1* gene and isolated central hypothyroidism were also described by Wassner et al. (2013).

The genetic abnormalities found in our reported patients suggest the lack of a functional protein. Both affected copies of the *PROP1* gene in patients lead to impairment of the main functional domain of the protein (frameshift, premature termination signal) and, therefore, production of truncated proteins with 164 and 112 amino acids residues, respectively. The pathogenic impact of the known c.150delA mutation has been well described and evidenced by numerous authors in the current literature (Fofanova et al. 1998; Riepe et al. 2001; Turton et al. 2005). Total abrogation of the gene function is reflected by the reported phenotype of the patients who were homozygous for the mutation.

The effect of a new change is somehow confusing. The phenotype of both probands clearly indicates genetic background, but uncommon manifestation suggests that an allele carrying the p.R112X change might potentially retain some residual protein activity. The functional core of the protein represents a DNA-binding homeodomain located between amino acids 68 and 130. The p.R112X mutation is placed directly in the homeodomain region and is terminating protein synthesis at the beginning of the third alpha helix (Fig. 4). Based on an in silico prediction of a reference and altered homeodomain orchestration, we noted that the residual amino acid sequence of p.R112X is identical to the corresponding part of the reference protein. The premature termination does not result in severe remodelling or misfolding of two proximal alpha helixes and, thus, this protein could potentially maintain some functionality. Because of the occurrence of a premature truncation codon at position 112, the NMD mechanism should also be regarded. However, the milder phenotype of the reported patients compared to carriers of i.e. homozygous c.150delA change indicates that this mechanism is not effective and, rather, does not result in substantial degradation of the abnormal c.334C>T transcript (if it even occurs at all). An influence of other compensatory genetic or environmental mechanisms should also be considered, particularly in regards to other reports presenting a varying range of clinical manifestations, depending on the type of mutation (Kelberman and Dattani 2007; Mody et al. 2002).

The newly discovered alteration of the *PROP1* gene is clinically relevant but the real spectrum of clinical consequences will be unveiled in patients with homozygous state. Both patients with gene mutations had definite thyroid hormones and probably GH and IGF-1 deficiency, starting in childhood; although they did not eventually attain a normal adult height, the presented skull dimensions and facial features were typical of non-affected adults. This may be explained by the modifying effect of thyroxine implemented early in the treatment on GH activity and its effect on bone growth (Do et al. 2015).

Similar clinical manifestation may appear in various *PROP1* gene defects. This case is addressed to an early central hypothyroidism as a first symptom in CPHD patients with different defects: p.R99X(Q), p.R112X and p.S109X (Tatsumi et al. 2004; Voutetakis et al. 2004b; Wassner et al. 2013). In a substantial portion of CPHD cases, abnormalities in *PROP1* and known CPHD-associated genes have not been identified. That implies an involvement of new genetic factors potentially contributing to the regulation of pituitary development and functioning, as an interesting target for further studies to be conducted. Furthermore, the constant improvement of genetic testing methods, i.e. whole-exome sequencing approach, that are more broadly employed for routine diagnostics will certainly facilitate the identification of CPHD cases at an earlier stage, even those with ambiguous course.
